# Antioxidant and Anti-Inflammatory Activities of Phenolic-Enriched Extracts of *Smilax glabra*

**DOI:** 10.1155/2014/910438

**Published:** 2014-11-11

**Authors:** Chuan-li Lu, Wei Zhu, Min Wang, Xiao-jie Xu, Chuan-jian Lu

**Affiliations:** ^1^The Second Institute of Clinical Medicine, Guangzhou University of Chinese Medicine, Guangzhou 510120, China; ^2^Guangdong Provincial Academy of Chinese Medical Sciences, Guangzhou 510120, China

## Abstract

*Smilax glabra* Roxb. has been used for a long time as both food and folk medicine. In the present study, phenolic-enriched extract of *S. glabra* (PEESG) was extracted with 70% ethanol and purified by HP-20 column chromatography. Its antioxidant and anti-inflammatory activities were evaluated by radical scavenging assay, reducing power determination, and lipopolysaccharide (LPS)-induced RAW264.7 cells assays, respectively. PEESG exhibited obviously scavenging capacity for DPPH and ABTS radicals, as well as significant reducing power for ferric ion. Particularly, PEESG (12.5–50 *μ*g/mL) showed a significantly higher efficiency for scavenging ABTS than that of ascorbic acid and no significant difference with ascorbic acid for DPPH scavenging. PEESG also possessed a significant suppression effect on proinflammatory mediators production, such as nitric oxide (NO), tumor necrosis factor-*α* (TNF-*α*), and interleukin-6 (IL-6), in LPS-induced RAW264.7 cells. In addition, the main ingredients of PEESG were identified using ultrahigh pressure liquid chromatography coupled to electrospray mass spectrometry (U-HPLC-ESI-MS). Seventeen components, including 5-O-caffeoylshikimic acid, neoastilbin, astilbin, neoisoastilbin, isoastilbin, engetin and isoengeletin were identified. These findings strongly suggest the potential of PEESG as a natural antioxidant and anti-inflammatory agent.

## 1. Introduction

*Smilax glabra* Roxb., a member of Liliaceae family, has been used for a long time as food and folk medicine in many countries. The rhizome of* S. glabra* in China is often consumed in soup, beneficial tea, and herbal medicine, while in Sri Lanka and Thailand it is often used in the preparation of medications for the treatment of cancer and skin conditions [1] and it is also used in other parts of the Asia, Arabian countries, and Europe for the treatment of many diseases.

Previous studies indicated that the major compositions of* S. glabra* were flavonoids [2] and phenylpropanoid esters [3]. In addition, terpenoids, mannose-binding lectin, and glycoproteins were also reported to exist in* S. glabra* [4, 5]. Various bioactivities of* S. glabra* have been demonstrated by* in vitro*/*in vivo* assays, which include antiproliferative [6], antiviral [4], anti-inflammatory [7], antihepatocarcinogenic [8], and immunomodulatory effects [9].

As mentioned above, the extract of* S. glabra* was rich in phenolics and flavonoids and possessed a potential utilization in health products. However, the antioxidant and anti-inflammatory activities of* S. glabra* were given insufficient attentions. Moreover, the main compositions, responsible for antioxidant and anti-inflammatory activities, were not yet fully elucidated. In the present research,* in vitro* assays were performed to investigate the antioxidant and anti-inflammatory activities of the phenolic-enriched extract of* S. glabra*, and a combination of UHPLC-DAD/ESI-MS/MS analysis was carried out to identify its main ingredients. Overall, the aim of the present study was to make a comprehensive understanding of* S. glabra*.

## 2. Materials and Methods

### 2.1. Chemicals

The RAW264.7 macrophage-like cell line from mice peritoneal macrophages was ordered from Laboratory Animal Center of Sun Yat-sen University (Guangzhou, China). Lipopolysaccharide (LPS), 2,2′-azino-bis(3-ethylbenzothiazoline-6-sulphonic acid) (ABTS), Folin-Ciocalteu's phenol reagent, 1,1-diphenyl-2-picrylhydrazyl (DPPH), and dimethyl sulfoxide (DMSO) were purchased from Sigma-Aldrich Co. (MO, USA). Dulbecco's modified Eagle's medium (DMEM) and fetal bovine serum (FBS) were purchased from Gibco (Gaithersburg, MD, USA). Ascorbic acid, gallic acid, rutin, diosgenin, and glucose were purchased from Aladdin Co. (Shanghai, China). Mouse IL-6 (E0079 m) and TNF-*α* (CSB-E04741 m) ELISA kits were purchased from Cusabio Biotech Co., Ltd. (Wuhan, China). Diaion HP-20 macroporous adsorption resin (250–600 *μ*m, 260 *Ǻ* ) was obtained from Mitsubishi Chemical Industries Ltd. (Tokyo, Japan). All other reagents and solvents were of analytical grade and all aqueous solutions were prepared using newly double-distilled water.

### 2.2. Preparation of PEESG

The rhizomes of* S. glabra* were purchased from Kangmei Pharmaceutical Co. Ltd. (Guangdong, China) in February 2013 (batch number 12120527) and were verified by Huang Zhi-hai in the Second Institute of Clinical Medicine, Guangzhou University of Chinese medicine (Guangzhou, China). The dried sample (100.00 g) was cut into thin slices and extracted with 70% ethanol (500 mL, at 60°C for 2 h) three times. The extracts were filtered with filter paper and collected. The combined filtrate was evaporated at 45°C (rotary evaporator, Buchi R-210) to give the ethanol extract of* S. glabra *(EESG, 16.73 g). EESG was sufficiently dissolved with 100 mL distilled water and centrifuged at 4000 rpm for 20 min. The supernatant was subjected to a Diaion HP-20 column (2 × 35 cm) with a gradient elution of ethanol-water: 0%, 60%, and 95% ethanol (v/v), and the fraction eluted by 60% ethanol was combined and evaporated to give PEESG (3.58 g).

### 2.3. Determination of Total Phenolic and Flavonoids Contents

Phenolic contents of EESG and PEESG were measured by the method described previously [10]. Briefly, 1.0 mL diluted solution of each sample was mixed with 0.5 mL Folin-Ciocalteu reagent (0.2 N) and stood at room temperature for 3 min, followed by the addition of 1.5 mL sodium carbonate solution (20%). After an incubation of 30 min, the absorbance was measured at 750 nm against water blank. A standard calibration curve was plotted using gallic acid (10–70 *μ*g/mL). Results were expressed as “mg of gallic acid equivalents per g of sample” (mg GAE/g sample).

Total flavonoids content was measured by a colorimetric assay described previously [11]. 2.0 mL sample solution (0.2 mg/mL) was put into a 10 mL flask, and 0.3 mL NaNO_2_ solution (5%) was added and mixed thoroughly. The solution was allowed to stand at room temperature for 6 min. Next, 0.3 mL Al(NO_3_)_3_ solution (10%) was added to the flask, mixed well, and kept at room temperature for 6 min. At last, 4 mL NaOH solution (4%) was added, mixed well, and kept at room temperature for 10 min. Absorbance at 510 nm was measured against water blank, and the concentration of flavonoids was estimated using calibration curves. Results were expressed as “mg of rutin equivalents per g of sample” (mg RE/g sample).

### 2.4. U-HPLC-DAD/ESI-MS/MS Analysis

In this study, U-HPLC-DAD/ESI-MS/MS analysis method was carried out to demonstrate and characterize the major constituents of PEESG. The analysis was performed on an Accela U-HPLC system (Thermo Fisher Scientific, San Jose, CA) coupled with LTQ Orbitrap XL hybrid mass spectrometer (Thermo Fisher Scientific, San Jose, CA, USA), fitted with an ESI source. Samples were separated on a reversed-phase Kinetex C18 column (100 mm × 2.10 mm, 1.7 *μ*m, Phenomenex Inc., USA) using a flow rate of 0.3 mL/min at 25°C. The mobile phase consisted of eluent A (methanol) and eluent B (aqueous formic acid solution, 0.2%, v/v). A gradient program was used for elution: 0–30 min, A from 15% to 95%, B from 85% to 5%. Analytes were determined by ESI-MS/MS selected reaction monitoring in the negative ion mode. The triple quadrupole MS and spray chamber conditions were gas temperature, 300°C; drying gas, nitrogen at 10 L/min; nebulizer pressure, 15 psi; sheath gas temperature, 250°C; sheath gas flow, nitrogen at 7 L/min; capillary voltage, 4 kV.

### 2.5. Antioxidant Property Assessment

#### 2.5.1. DPPH Radical Scavenging Assay

DPPH assay was carried out using a modified method described previously [12]. Briefly, 3.0 mL sample solution with various concentrations (200–12.5 *μ*g/mL) was mixed with 2.0 mL DPPH solution (0.2 mM, dissolved in ethanol). After being incubated for 30 min in the dark at room temperature, the absorbance of the mixture against blank was determined at 520 nm. The DPPH radical scavenging activity was calculated as percentage of inhibition according to the following equation:

(1) DPPH  radical  scavenging  ratio % =1−AS−AS0A0×100%,

where *A*_0_ is the absorbance of a blank treatment group, while *A*_*S*_ is the absorbance of a sample treatment group and *A*_*S*0_ is the absorbance of a sample background. All measurements were performed in triplicate and ascorbic acid was used as a positive standard.

#### 2.5.2. ABTS Radical Scavenging Assay

Scavenging capacity of ABTS radicals was carried out according to the method described previously [13]. The ABTS^•+^ was prepared by mixing an ABTS stock solution (7 mM in water) with 2.45 mM potassium persulfate. This mixture was kept still for 16 h at room temperature in the dark. The ABTS^•+^ working solution was obtained by diluting the stock solution in methanol to an absorbance of 0.7 ± 0.10 at 747 nm. 0.5 mL appropriately diluted sample was added to 5.0 mL ABTS^•+^ working solution and mixed thoroughly. The reaction mixture was kept at room temperature in the dark for 6 min, and the absorbance was recorded at 747 nm. ABTS radical scavenging activity was calculated as follows:

(2) ABTS  radical  scavenging  ratio % =1−AS−AS0A0×100%,

where *A*_0_ is the absorbance of a blank treatment group, while *A*_*S*_ is the absorbance of a sample treatment group and *A*_*S*0_ is the absorbance of a sample background. All measurements were performed in triplicate and ascorbic acid was used as a positive standard.

#### 2.5.3. Reducing Power Assay

The reducing power of the samples was determined as previously described [14]. Potassium ferricyanide (2.5 mL, 10 mg/mL) was added to samples in phosphate buffer (2.5 mL, 200 mM, pH 6.6) and the mixture was incubated at 50°C for 20 min. Trichloroacetic acid (2.5 mL, 100 mg/mL) was added to the mixture, which was then centrifuged at 3,000 rpm for 10 min. The supernatant (2.5 mL) was mixed with distilled water (2.5 mL) and ferric chloride (0.5 mL, 1.0 mg/mL), and then the absorbance was read at 700 nm. Higher absorbance of the reaction mixture indicated greater reducing activity.

### 2.6. Anti-Inflammatory Effects Assay

#### 2.6.1. Cell Culture

The RAW264.7 cells were maintained in DMEM supplemented with 10% fetal bovine serum, 100 U/mL penicillin, and 100 *μ*g/mL streptomycin at 37°C in a humidified atmosphere of 5% CO_2_. Cells in log phase were used for experiments.

#### 2.6.2. MTT Cell Viability Assay

RAW264.7 cells in log phase were seeded in 96-well plates (3 × 10^3^ cells/well) and incubated for 12 h and then treated with different concentrations of samples. After 24 h, 10 *μ*L MTT (5 *μ*g/mL) was added to each well, and the cultures were incubated for an additional 4 h. The medium was removed carefully, and 150 *μ*L DMSO was added to dissolve formazan crystals. The plates were shaken for 10 min, and then the optical density (OD) value was detected at 490 nm.

#### 2.6.3. Measurement of Nitric Oxide/Nitrite

This assay was carried out according to the methods described previously [15]. The accumulation of nitrite, an indicator of NO production in the culture medium, was measured with the Griess regent (0.1% N-(1-naphthyl) ethylenediamine dihydrochloride, 1% sulfanilamide, and 2.5% H_3_PO_4_). RAW264.7 cells were seeded into 96-well culture plates (3 × 10^5^ cells/well). After 24 h incubation, cells were stimulated by LPS (1 *μ*g/mL) with or without different concentrations of samples for 24 h. Subsequently, the supernatant (100 *μ*L) was harvested, mixed with an equal volume of Griess reagent, and allowed to stand for 15 min at room temperature in the dark. Absorbance at 540 nm of the reaction was monitored with a microplate reader. The concentration of NO was evaluated through a standard curve prepared according to the producer.

#### 2.6.4. Enzyme-Linked Immunosorbent Assay for IL-6 and TNF-*α* Cytokines Detection

Cells were seeded at a density of 5 × 10^5^ cells/well in 24-well plates. After 24 h incubation, cells were stimulated by LPS (1 *μ*g/mL) with or without different concentrations of samples for 24 h. The release of IL-6 and TNF-*α* in supernatant was determined using ELISA kits according to the manufacturer's instructions.

### 2.7. Statistical Analysis

All assays were carried out in triplicate or quintuplicate and the mean values were counted. The data were subjected to analysis of variance (ANOVA) for comparing three or more groups, where Student's *t*-test was used to assess the differences between means values. A significant difference was assumed at a level of *P* < 0.05.

## 3. Results and Discussion

### 3.1. Total Phenolic/Flavonoid Content

In the present study, the total phenolic content and total flavonoid content of PEESG are, respectively, 563.88 ± 15.00 (mg GAE/g sample) and 1147.82 ± 60.19 (mg RE/g sample), which are obviously higher than those of EESG (Table 1). In previous reports, total phenolic level of methanol and water extracts from Rhizoma Smilacis Glabrae were correspondingly 152.28 ± 10.57 and 29.41 ± 3.14 mg GAE/g sample [16], while total phenolic content and total flavonoids content of 60% ethanol extract of* S. glabra* were 262.6 ± 12.7 mg GAE/g sample and 203.4 ± 9.1 mg RE/g sample, respectively [17]. These results are consistent with our findings.

### 3.2. Identification of Phenols in PEESG

**Figure 1:**
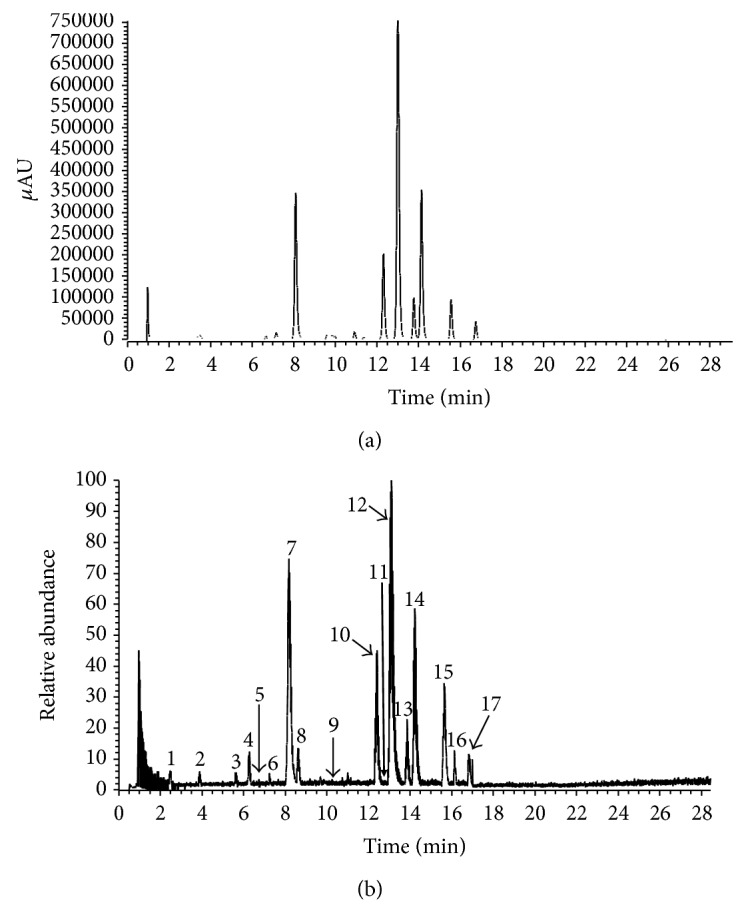
UHPLC-PDA/ESI-MS analysis of the PEESG. (a) UHPLC-PDA (295 nm) chromatography. (b) ESI-MS (negative) total ion current profile.

Data from the LC-DAD-ESI/MS were used to identify the phenolics in PEESG. DAD chromatogram (295 nm) and total ion current (TIC) are shown in Figure 1. The retention times (*t*_*R*_), UV *λ*_max⁡_ values, and the molecular ions of the phenolics are listed in Table 2 for each peak. By referencing to the reported data of their chromatograms, full UV/vis and mass spectra for each peak, 17 compounds were characterized, including 8 phenolics and 9 flavonoids. Among these constituents, 5-O-caffeoylshikimic acid (peak 7), neoastilbin (peak 10), astilbin (peak 12), neoisoastilbin (peak 13), isoastilbin (peak 14), engetin (peak 15), and isoengeletin (peak 15) were the major components of PEESG.

### 3.3. Antioxidant Activity

Phenolic compounds are a major class of bioactive components, which have been demonstrated to be better antioxidants* in vitro* than ascorbic acid [18]. Polyphenols possess the ideal chemistry for antioxidant activity because they have high reactivity as hydrogen or electron donors and also they are capable of chelating metal ions [19]. Flavonoids, one of the major polyphenolic constituents of plants, were found ubiquitously in plant kingdom. They were known for their efficient radical scavenging activity owing to their hydroxyl group at various positions [20].

**Figure 2:**
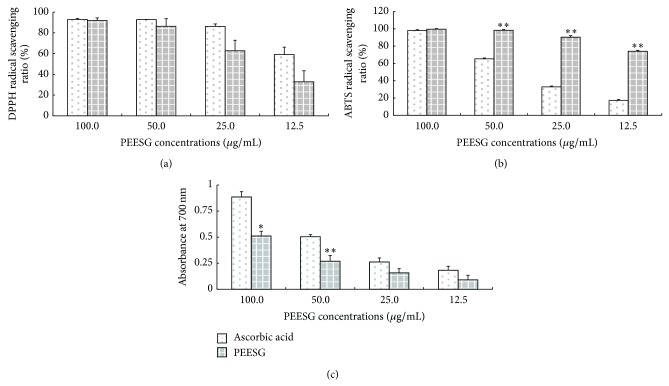
Antioxidant activity of PEESG. The antioxidant activity of PEESG was evaluated by DPPH radical scavenging assay (a), ABTS radical scavenging assay (b), and reducing power evaluation (c). Each experiment was repeated three times, and results represent the mean ± SD. Ascorbic acid was employed as a positive control. ∗ and ∗∗ mean *P* < 0.05 and *P* < 0.01 correspondingly compared with ascorbic acid group.

In the present study, radical scavenging capability assays (DPPH and ABTS) and reducing power determination were carried out to evaluate the antioxidant ability of PEESG, and results were displayed in Figure 2. PEESG showed a dose-dependent scavenging activity for both DPPH (Figure 2(a)) and ABTS radicals (Figure 2(b)), as well as remarkable reducing power (Figure 2(c)). DPPH^•^ and ABTS^•^ are stable free radicals, which are frequently used for evaluation of a radical scavenging activity of natural compounds [21]. In the present study, PEESG exhibited no different scavenging capability of DPPH from that of ascorbic acid (*P* > 0.05) and a significantly higher scavenging capability of ABTS than that of ascorbic acid (*P* < 0.01). At the concentration of 100 *μ*g/mL, PEESG could scavenge almost 91.91% of DPPH radicals.

In Zhang's studies [16], methanol, water extract of* S. glabra*, and supernatant fraction showed dose-dependent antioxidant activity but polysaccharide did not show any antioxidant activity. Cai et al. evaluated the antioxidant activity of 112 traditional Chinese medicinal plants, and results indicated that the Trolox equivalent antioxidant capacity of methanol and water extracts of* S. glabra* was 137.3 and 75.5 *μ*mol Trolox/100 g sample, respectively, [22]. Furthermore, astilbin, considered as the main component of* S. glabra*, has been demonstrated to possess potential antioxidant property [16, 23]. Epicatechin and catechin were also well known antioxidants from widely consumed tea leaf [24].

### 3.4. Anti-Inflammatory Activity

Macrophages are extraordinarily versatile cells, playing an essential role in a host's defense against bacterial infection by nature of their phagocytic, cytotoxic, and intracellular killing capacities [25]. LPS, the structural component of the Gram-negative bacteria outer cell well, is a potent initiator of the inflammatory response during most bacterial infections [26]. In response to LPS, the peritoneal macrophages readily secrete various inflammatory mediators including IL-1, IL-6, TNF-*α*, and NO [27]. Therefore, the suppression of TNF-*α*, IL-6, and NO production can be a very important therapeutic target in the development of anti-inflammatory agents.

In the present study, the anti-inflammatory activity of PEESG was evaluated on LPS-stimulated RAW264.7 cells model, and results were presented in Table 3, which showed that the accumulation of NO, IL-6, and TNF-*α* in LPS-stimulated groups was remarkably higher than that of control group (*P* < 0.01). Treatment with various concentrations (40, 8, and 1.6 *μ*g/mL) of PEESG showed a significant dose-dependent inhibition for LPS-induced production of NO, IL-6, and TNF-*α* (*P* < 0.05). In addition, cells viability assay suggested that PEESG at test concentrations displayed no effect on RAW264.7 cell viability.

Previous reports indicated that the aqueous extract from Rhizoma Smilacis Glabrae could selectively inhibit the effector phase of delayed-type hypersensitivity without suppressing humoral immune response [28], and the water extract of* S. glabra* exhibited a remarkable inhibition against both primary and secondary hind paw swelling of adjuvant arthritis in rats [29]. Furthermore, the aqueous extract of* S. glabra* exhibited an improvement on adjuvant arthritis through downregulating overactivated macrophages and upregulating the dysfunctional T lymphocytes during the later phase of arthritis [9]. Additionally, many formulations including* S. glabra* were proved to possess anti-inflammatory activity* in vivo* and* in vitro* [7, 30].

Astilbin, isolated from* Engelhardia roxburghiana*, exhibited significant inhibitory effects on the activity of LPS-stimulated mouse J774A.1 macrophage cells [31]. Epicatechin has also been demonstrated to have a systemic anti-inflammatory activity. de Paula Vasconcelos et al. investigated the pathway of epicatechin in the prevention and treatment of intestine inflammation in acute and chronic rat models, and they confirmed that epicatechin as an antioxidant could reduce the lesion and stimulate tissue healing and was also conducive to preventing intestine inflammation [32].

## 4. Conclusions

Taken together, the present study demonstrated that PEESG possesses valuable antioxidant and anti-inflammatory activities. The results in the present study support the pharmacological basis of PEESG for the treatment of inflammatory illnesses. Further studies are still needed to elucidate the mechanism and therapeutic potential of PEESG.

## Figures and Tables

**Table 1:** Total phenolic and flavonoid contents of EESG and PEESG.

	EESG	PEESG
Total phenolics content (mg GAE/g sample)	208.45 ± 3.74	563.88 ± 15.00^**^
Total flavonoids content (mg RE/g sample)	420.26 ± 125.67	1147.82 ± 60.19^*^

Each value represents the mean ± SD (*n* = 3). ∗ and ∗∗ mean *P* < 0.01 and *P* < 0.001 correspondingly compared with EESG group.

**Table 2:** Characterization of major constituents of ethanol extract of *S. glabra* by UHPLC-DAD-MS/MS.

Number	*t*_*R*_ (min)	*λ*_max_ (nm)	Observed mass	Calculated mass	Error (ppm)	Formula [M–H]^−^	LC-MS^2^ data	Constituents
1	2.44	280	255.0497	255.0499	−0.781	C_11_H_11_O_7_	193.0504, 179.0349, 165.0557	Phenolic acid derivatives
2	3.87	252, 285	289.0702	289.0707	−1.54	C_15_H_13_O_6_	245.0816, 205.0504, 179.0348	Epicatechin
3	5.61	227, 283	239.0549	239.0550	−0.52	C_11_H_11_O_6_	221.0456, 199.8137, 195.1390, 179.0348, 177.0556, 149.0608	Phenolic acid derivatives
4	6.24	227, 280	289.0702	289.0707	−1.23	C_15_H_13_O_6_	245.0816, 205.0504, 179.0348	Catechin
5	6.72	276	335.0753	335.0761	−2.46	C_16_H_15_O_8_	291.0871, 179.0349, 135.0450	3-O-Caffeoylshikimic acid
6	7.24	253, 285	335.0754	335.0761	−2.10	C_16_H_15_O_8_	291.0871, 179.0349, 135.0450	4-O-Caffeoylshikimic acid
7	8.17	254, 326	335.0757	335.0761	−1.30	C_16_H_15_O_8_	291.0871, 179.0349, 135.0450	5-O-Caffeoylshikimic acid
8	8.62	255, 283	451.1009	451.1024	−2.79	C_24_H_19_O_9_	341.0660	Cinchonain Ia
9	9.78	285	303.0492	303.0499	−1.91	C_15_H_11_O_7_	285.0401	Taxifolin
10	12.39	290	449.1067	449.1078	−2.56	C_21_H_21_O_11_	431.0977, 323.0768, 303.0507, 285.0401, 151.0037	Neoastilbin
11	12.82	209, 284	451.1009	451.1024	−3.19	C_24_H_19_O_9_	341.0660	Cinchonain Ib
12	13.10	241, 290	449.1069	449.1078	−2.09	C_21_H_21_O_11_	431.0977, 323.0768, 303.0507, 285.0401, 151.0037	Astilbin
13	13.82	294	449.1065	449.1078	−3.02	C_21_H_21_O_11_	431.0977, 323.0768, 303.0507, 285.0401, 151.0037	Neoisoastilbin
14	14.22	251, 295	449.1067	449.1078	−2.62	C_21_H_21_O_11_	431.0977, 323.0768, 303.0507, 285.0401, 151.0037	Isoastilbin
15	15.67	292	433.1115	433.1129	−3.33	C_21_H_21_O_10_	287.0558, 269.0452, 259.0608	Engeletin
16	16.16	281	451.1010	451.1024	−3.19	C_24_H_19_O_9_	341.0660	Phenolic acid derivatives
17	16.82	293	433.1117	433.1129	−2.92	C_21_H_21_O_10_	287.0558, 269.0452, 259.0608	Isoengeletin

**Table 3:** Effect of EESG and PEESG on secretions of NO, IL-6, and TNF-a in LPS-induced RAW264.7 cells.

Groups	Dose (*μ*g/mL)	NO (ng/mL)	IL-6 (pg/mL)	TNF-a (pg/mL)
Control		0.229 ± 0.083	9.497 ± 3.402	37.995 ± 16.422

LPS	0.1	9.308 ± 0.307^##^	115.100 ± 31.988^#^	3759.263 ± 496.642^##^

LPS + DXM	0.25	3.124 ± 0.173^***^	23.531 ± 8.717^**^	318.154 ± 64.996^***^

PEESG	40	5.298 ± 0.355^***^	37.900 ± 12.299^*^	617.741 ± 197.251^***^
	8	7.529 ± 0.410^***^	65.971 ± 10.672^*^	1730.369 ± 325.747^**^
	1.6	8.106 ± 0.323^**^	84.457 ± 16.659	2682.134 ± 349.289^*^

Cells were treated with LPS (0.1 *μ*g/mL) for 24 h in the absence or presence of samples (1.6, 8.0, and 40 *μ*g/mL). The data were presented as mean ± SD (*n* = 5). Dexamethasone (DXM) was employed as a positive control. # and ## mean *P* < 0.01 and *P* < 0.001 correspondingly compared with control group while ∗, ∗∗, and ∗∗∗ mean *P* < 0.05, *P* <0.01, and *P* < 0.001 correspondingly compared with LPS group.

## Practical Application

Our findings demonstrate the potential of PEESG as a natural antioxidant and anti-inflammatory agent.
